# Supplementary material for: Single-cell and Spatial Omics Reveals Region-Specific Plasticity and Therapeutic Vulnerabilities in Metastatic High-Risk Neuroblastoma
Source: bioRxiv. 2025 Oct 20:2025.10.19.681936. Preprint. [Version 1] doi: 10.1101/2025.10.19.681936 (PMC12633255; doi:10.1101/2025.10.19.681936)
Supplement: 1 [file NIHPP2025.10.19.681936V1-supplement-1.pdf]

bioRxiv preprint doi: https://doi.org/10.1101/2025.10.19.681936; this version posted October 20, 2025. The copyright holder for this preprint (which was not certified by peer review) is the author/funder, who has granted bioRxiv a license to display the preprint in perpetuity. It is made available under aCC-BY 4.0 International license.

Supplemental figure legends:

Supplementary Fig. S1. Comparison of cellular landscapes of high-risk primary NB and metastatic lesions resected post-treatment.

a. UMAP visualization of single-cell transcriptome data of 92,227 cells from primary adrenal glands and lymph node metastatic sites of NBs from patients post-treatment. Colors represent assigned cell types within NB tumors.
b. Side-by-side UMAP visualization of post-treatment NBs at adrenal glands (n=6; 50,922 cells) and at lymph node metastases (n=5; 41,305 cells). Red boxes denote tumor cell phenotypes in the NB tumor.
c. A summary of cell type proportions in post-treatment NBs at primary and metastatic sites.

Supplementary Fig. S2. Differential gene marker expression in primary and lymph node metastatic sites.

a. Differentially expressed genes of early NB progenitor populations (left) and tumor cells (right) from post-treatment NBs at primary and metastatic sites. Color labels indicate visual outliers on a scatter plot in primary and metastatic NBs.
b. UMAP plots of marker genes within NB clusters based on Slingshot trajectories.

Supplementary Fig. S3. Cell states in NB tumors are heterogeneous.

a. UMAP visualization of integrated scRNA-seq datasets of tumor cell phenotypes in primary tumors of treatment-naïve high-risk NB patients and in primary and metastatic tumors of post-treatment high-risk NB patients. Colors represent NB cell tumor types.
b. UMAP visualizations of tumor cell subclusters (red rectangles in (Figure 1c) in primary tumors of treatment-naïve patients (8,905 cells), primary tumors of post-treatment patients (25,434 cells), and lymph node metastases of post-treatment patients (23,301 cells). Cells are colored according to tumor cell type.
c. Proportions of NB-derived cell subtypes in primary tumors from treatment-naïve patients, primary tumors of post-treatment patients, and lymph node metastases of post-treatment patients.
d. Marker gene expression in NB-derived cell clusters from NB tumors. The color represents scaled average expression of marker genes in each cell type, and the size indicates the proportion of cells that express marker genes.

Supplementary Fig. S4. CNVs inferred from snRNA-seq of NB tumor cells from primary and metastatic lesions.

CNVs detected in snRNA-seq data in post-treatment primary (n=6; left panel) and metastatic samples (n=5; right panel) with cut-off for the minimum average read counts per gene among reference stromal and immune cells set at 0.1. Each row corresponds to a cell, ordered by tumor cell phenotype, and clustered within each tumor by CNV patterns.

Supplementary Fig. S5. NB metastatic cell states have immune and mesenchymal signatures distinct from embryonic sympathoblasts.

a. Heatmap of cell proportions in tumor cell states in post-treatment NBs at primary (n=9) and metastatic sites (n=7) relative to those in developing lineages in sympathoadrenal regions during human embryogenesis from post-conception weeks 6-14 (GSE147821) [15] determined using CIBERSORTx deconvolution of scRNA-seq data.
b. Predicted proportions of indicated cell types in primary and metastatic NBs. *P < 0.05 and **P < 0.01, multiple t tests using the Holm-Sidak method. Data are means ± SEM.
c. UMAP visualization of integrated scRNA-seq datasets of human embryogenesis lineages (GSE147821; n=134,491), post-treatment primary NB (n= 50,922 cells), and metastatic NB (n= 41,305 cells).
d. Side-by-side UMAP visualizations of human embryogenesis lineages, post-treatment primary NB, and metastatic NB. Red rectangles denote the sympathoadrenal lineages in fetal embryo and NB-derived

bioRxiv preprint doi: https://doi.org/10.1101/2025.10.19.681936; this version posted October 20, 2025. The copyright holder for this preprint (which was not certified by peer review) is the author/funder, who has granted bioRxiv a license to display the preprint in perpetuity. It is made available under aCC-BY 4.0 International license.

tumor cell phenotypes in NBs.

e. Cell type proportions in human embryogenesis lineages (Embryo) and individual post-treatment primary and metastatic samples.
f. Marker gene expression in embryogenesis lineages and NBs from primary and metastatic tumors. The color represents scaled average expression of marker genes in each cell type, and the size indicates the proportion of cells that express the marker gene. SCP, Schwann cell precursors; HSC, hematopoietic stem cells.

Supplementary Fig. S6. Integrative single-cell transcriptomic and epigenomic profiles reveal accessible genes in primary and metastatic NBs.

a. Fragment size distributions of four primary and four metastatic NB scATAC-seq datasets.
b. Transcription-start-site (TSS) enrichment scores of primary and metastatic NBs.
c. Activity scores for indicated genes across cell types in primary and metastatic NBs.
d. Genome browser tracks of co-accessibility at adrenergic, mesenchymal, stem cell, and metastasis marker gene loci in indicated cell types from NBs.

Supplementary Fig. S7. Cytokine genes are differentially expressed in lymphocytes of primary and metastatic NBs.

a. Expression of indicated genes in lymphocyte clusters.
b. Expression of cytokine genes primary and metastatic NBs.

Supplementary Fig. S8. Cell-type-specific communication maps of primary NBs and lymph node metastases.

a. Dot plots of outgoing (top) and incoming (bottom) signaling patterns of spatially proximal cell-cell communications between indicated cell types in primary NBs. Dot size is proportional to the contribution score computed from pattern recognition analysis using CellChat v2 [53]. Higher contribution scores indicate more enriched signaling pathway in the corresponding cell group.
b. Dot plots of outgoing (top) and incoming (bottom) signaling patterns of spatially proximal cell-cell communications between indicated cell types metastatic sites.

Supplementary Fig. S9. Inhibition of protein translation machinery and nuclear expert synergistically halts NB cell growth.

a. Representative immunohistochemistry images of peripheral nerve, primary NB, and metastases stained for EIF4A, EIF4G, and EIF4E. Scale bars, 100 µm.

b, c, Growth curves of human NB-derived cell lines CHLA15 (primary), CHLA20 (relapsed), and SK-N-BE2 (relapsed) treated with b) didesmethylrocagamide (DDR) or c) rocaglamide (Roc) (right). Cell viability was estimated as a percentage of the DMSO vehicle control. Graphs depict the means and SEMs from 2 or 3 independent experiments. Estimated IC_{50} values for each cell line are included in graph legend.

d, Percent inhibition of growth of NB tumor cells as a function of Roc (left) and selinexor (right) concentrations. Red circles are means of 4 replicates.

e, 2D dose response matrix showing the mean percent inhibition ± SD for each selinexor/Roc concentration combination (n=4).

f. 2D matrix array (upper) and 3D contour plot (lower) of highest single agent (HSA) synergy scores calculated using SynergyFinder Plus [92].

bioRxiv preprint doi: https://doi.org/10.1101/2025.10.19.681936; this version posted October 20, 2025. The copyright holder for this preprint (which was not certified by peer review) is the author/funder, who has granted bioRxiv a license to display the preprint in perpetuity. It is made available under aCC-BY 4.0 International license.

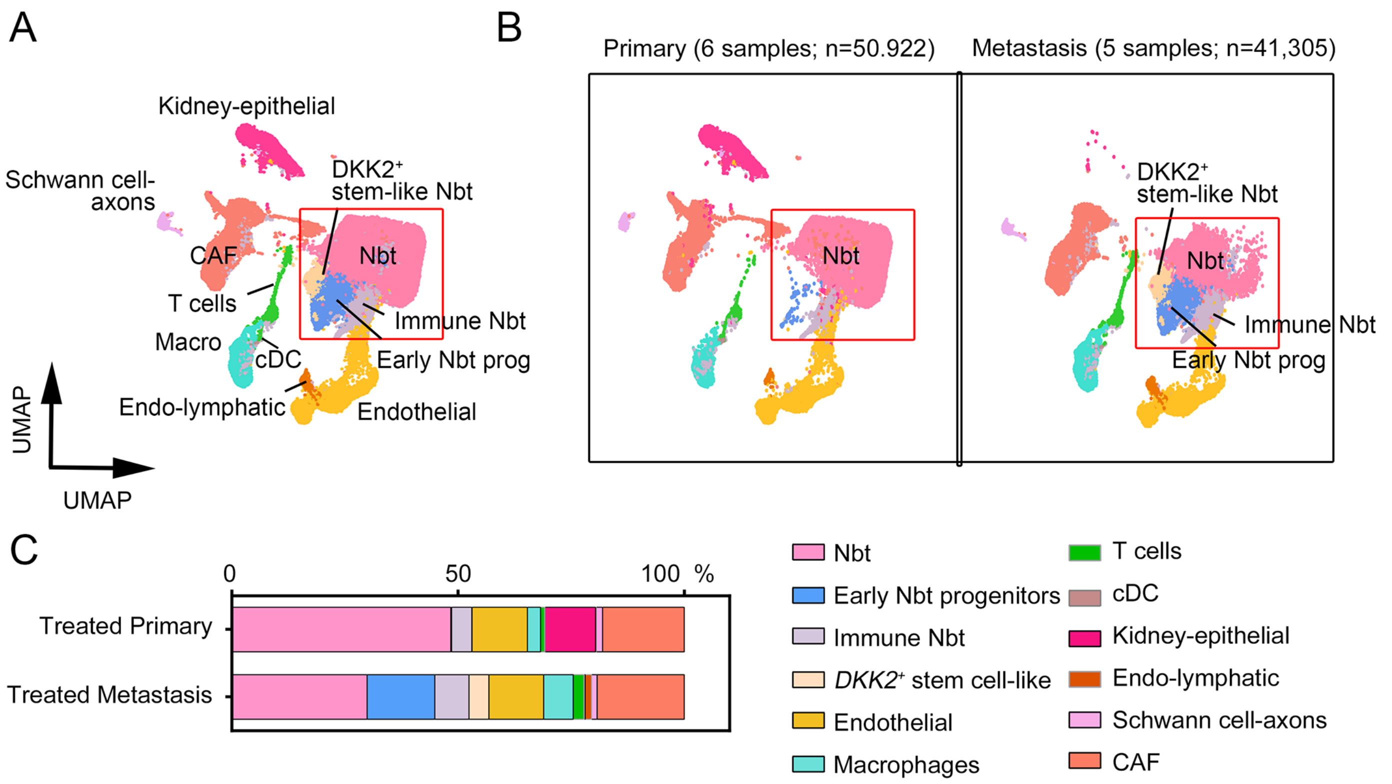

Figure S1

bioRxiv preprint doi: https://doi.org/10.1101/2025.10.19.681936; this version posted October 20, 2025. The copyright holder for this preprint (which was not certified by peer review) is the author/funder, who has granted bioRxiv a license to display the preprint in perpetuity. It is made available under aCC-BY 4.0 International license.

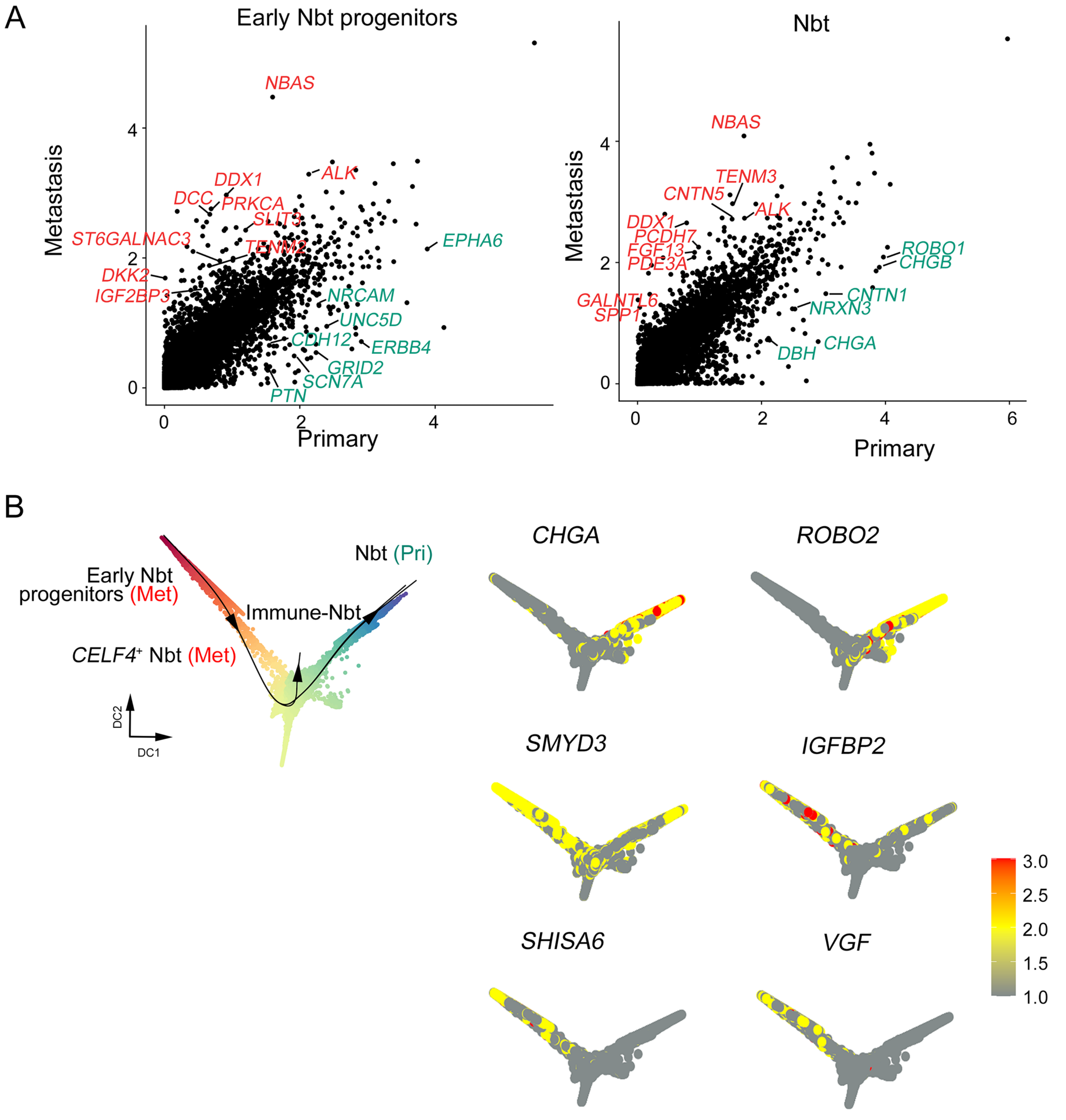

Figure S2

bioRxiv preprint doi: https://doi.org/10.1101/2025.10.19.681936; this version posted October 20, 2025. The copyright holder for this preprint (which was not certified by peer review) is the author/funder, who has granted bioRxiv a license to display the preprint in perpetuity. It is made available under aCC-BY 4.0 International license.

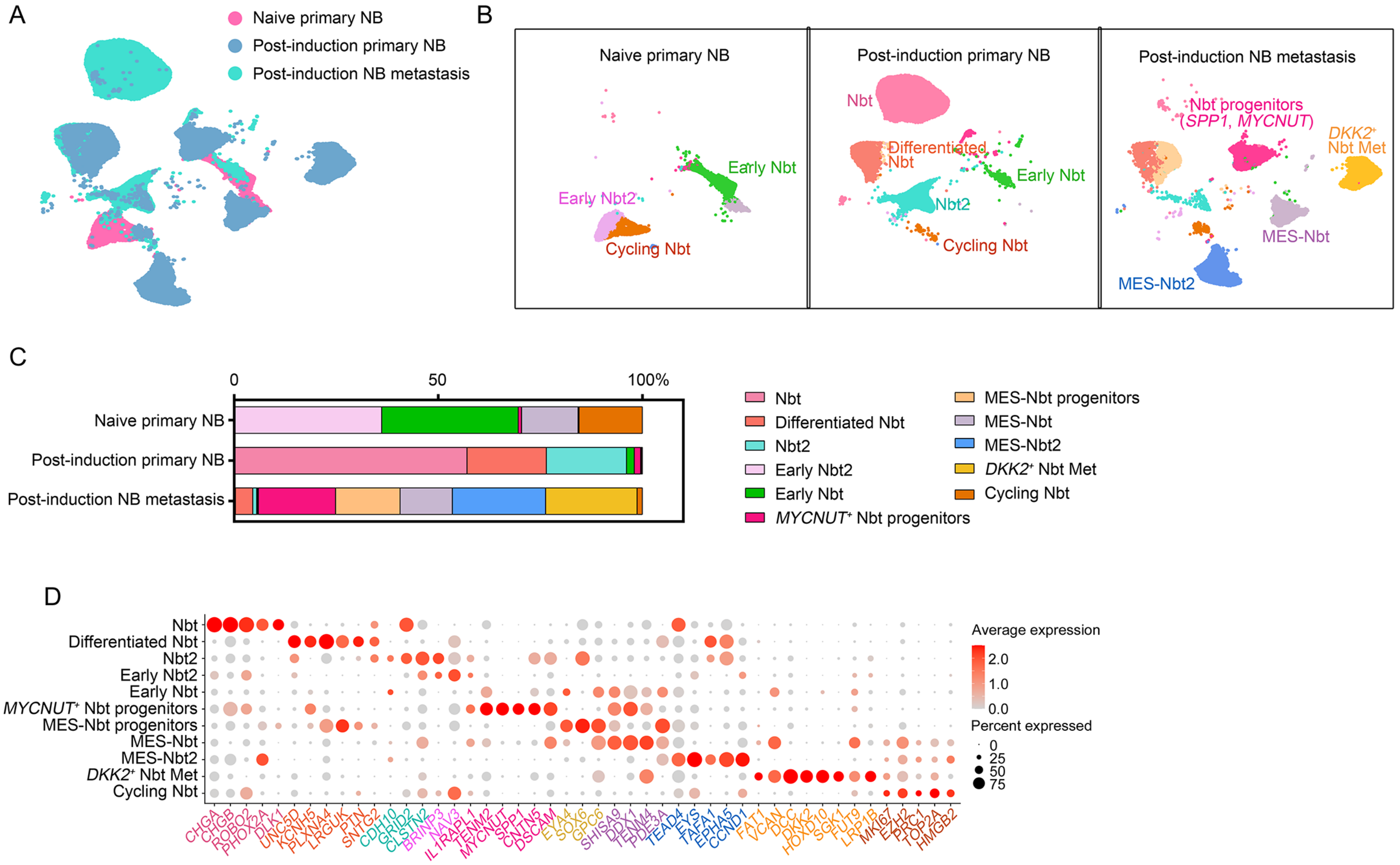

Figure S3

bioRxiv preprint doi: https://doi.org/10.1101/2025.10.19.681936; this version posted October 20, 2025. The copyright holder for this preprint (which was not certified by peer review) is the author/funder, who has granted bioRxiv a license to display the preprint in perpetuity. It is made available under aCC-BY 4.0 International license.

Post-induction NB at Primary Site (n=6 samples)

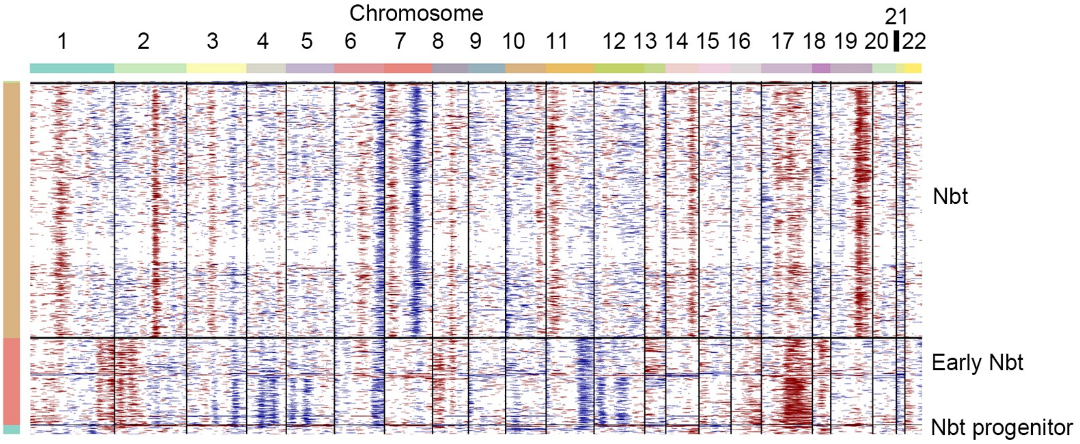

Post-induction NB at Metastatic Site (n=5 samples)

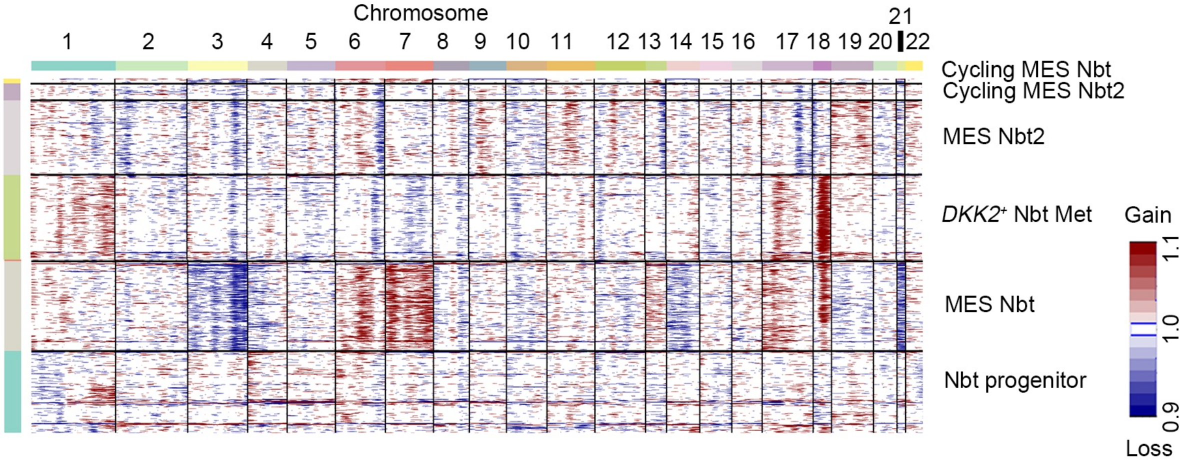

Figure S4

bioRxiv preprint doi: https://doi.org/10.1101/2025.10.19.681936; this version posted October 20, 2025. The copyright holder for this preprint (which was not certified by peer review) is the author/funder, who has granted bioRxiv a license to display the preprint in perpetuity. It is made available under aCC-BY 4.0 International license.

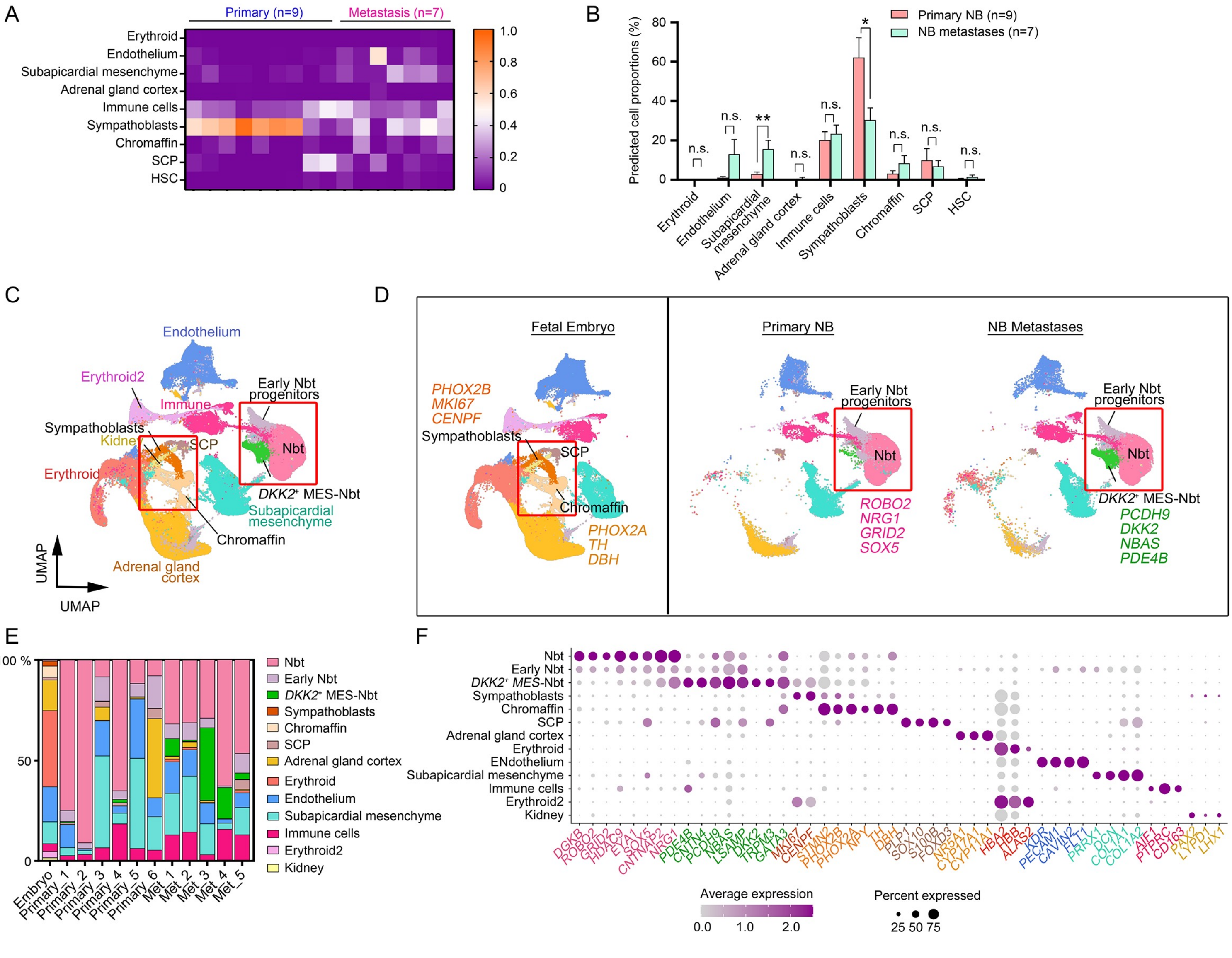

Figure S5

bioRxiv preprint doi: https://doi.org/10.1101/2025.10.19.681936; this version posted October 20, 2025. The copyright holder for this preprint (which was not certified by peer review) is the author/funder, who has granted bioRxiv a license to display the preprint in perpetuity. It is made available under aCC-BY 4.0 International license.

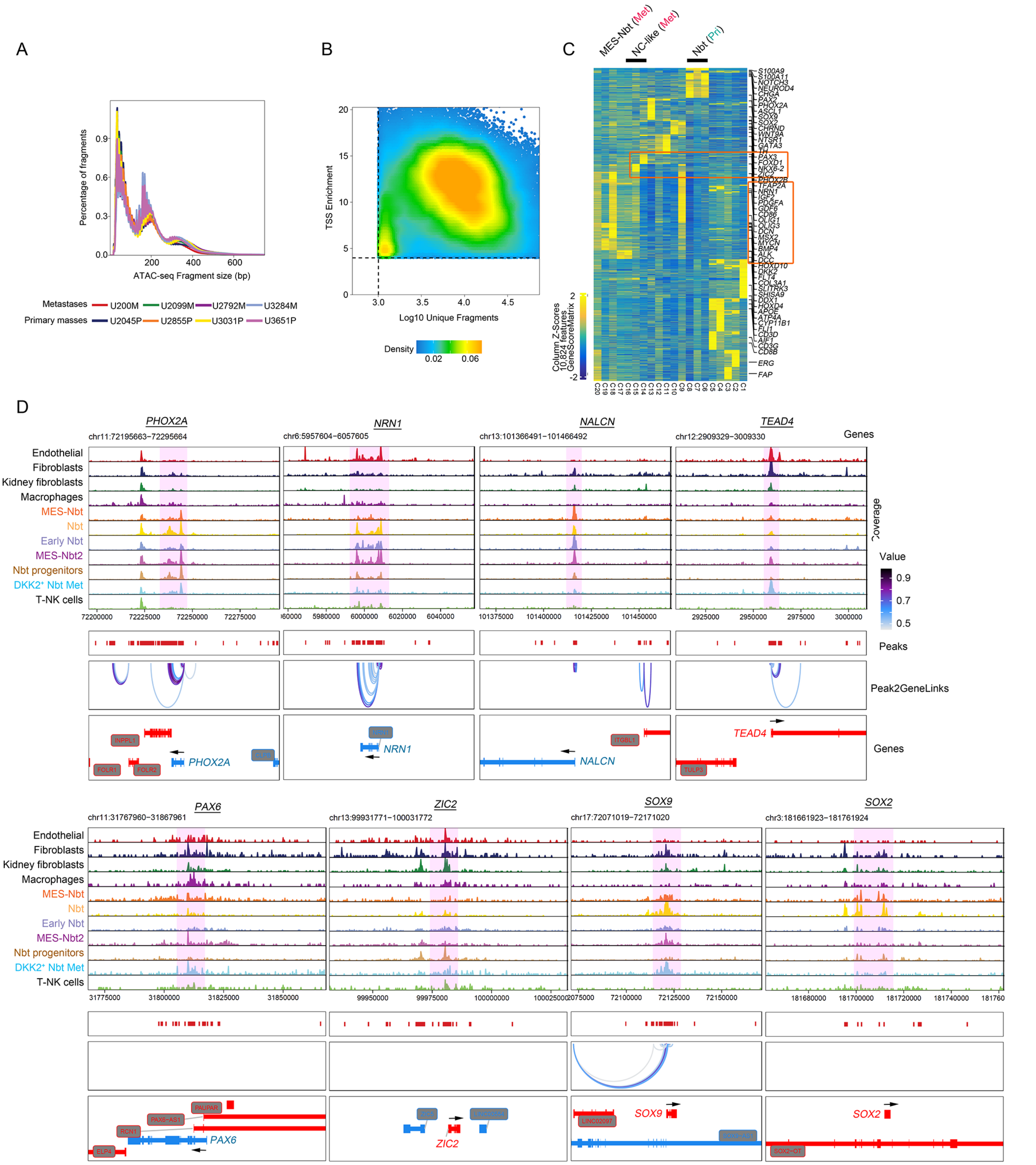

Figure S6

bioRxiv preprint doi: https://doi.org/10.1101/2025.10.19.681936; this version posted October 20, 2025. The copyright holder for this preprint (which was not certified by peer review) is the author/funder, who has granted bioRxiv a license to display the preprint in perpetuity. It is made available under aCC-BY 4.0 International license.

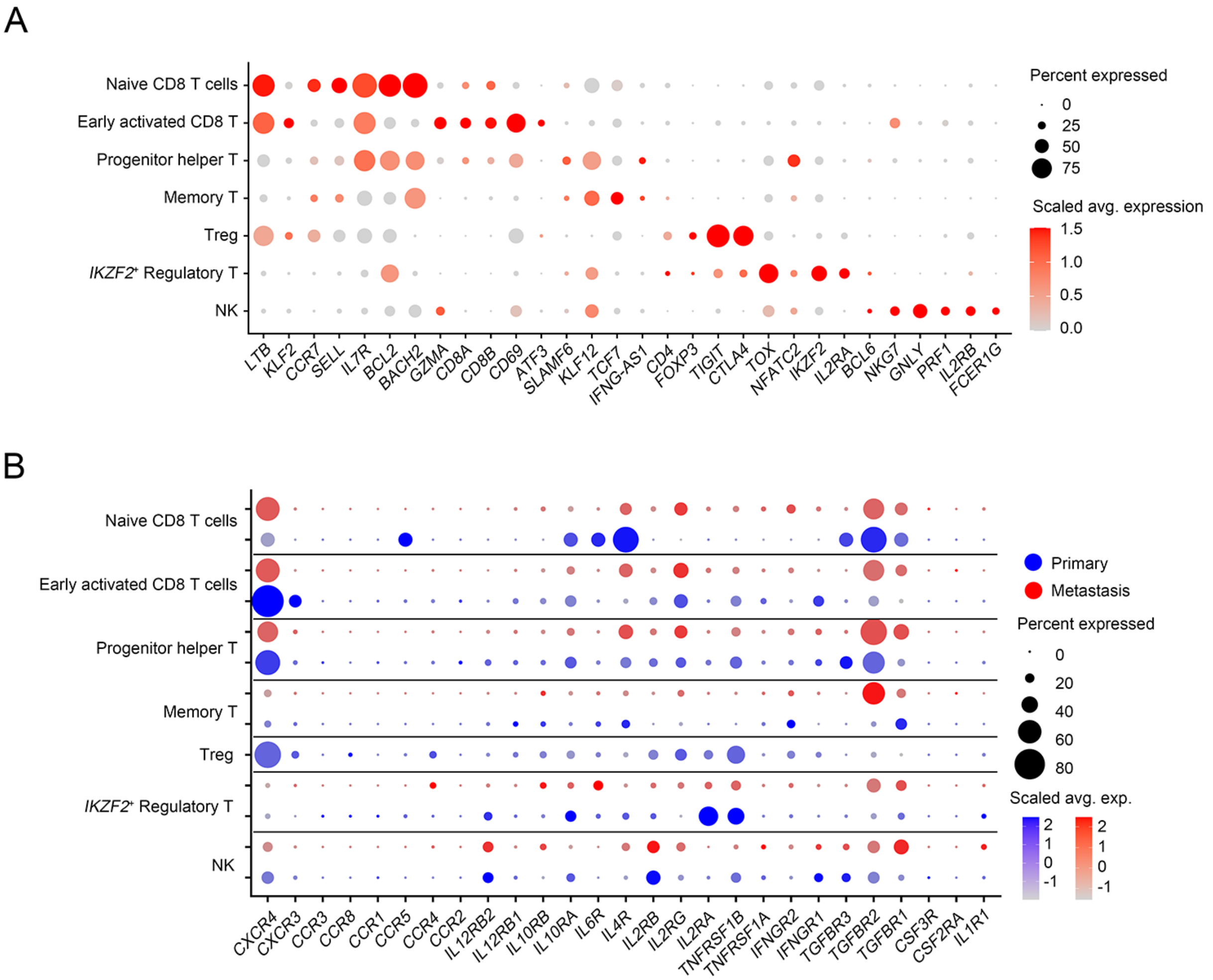

Figure S7

bioRxiv preprint doi: https://doi.org/10.1101/2025.10.19.681936; this version posted October 20, 2025. The copyright holder for this preprint (which was not certified by peer review) is the author/funder, who has granted bioRxiv a license to display the preprint in perpetuity. It is made available under aCC-BY 4.0 International license.

A

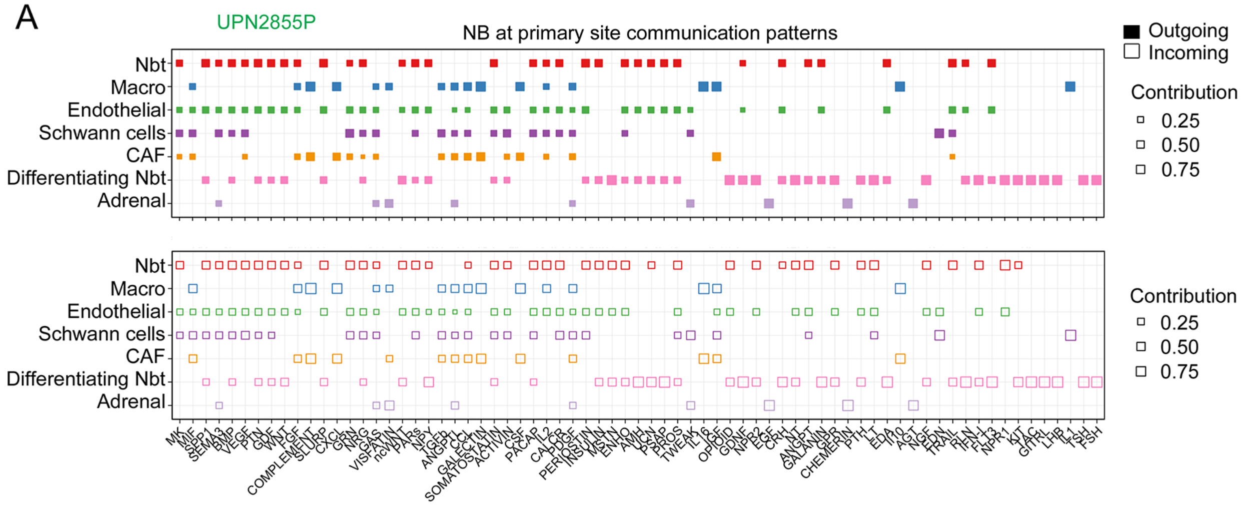

B

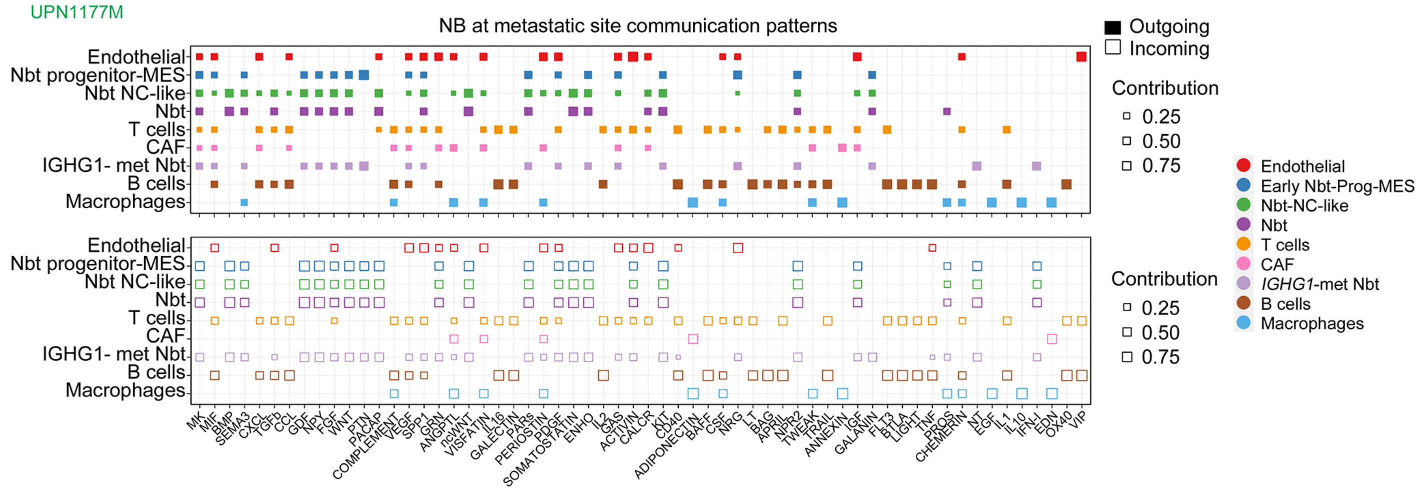

Figure S8

bioRxiv preprint doi: https://doi.org/10.1101/2025.10.19.681936; this version posted October 20, 2025. The copyright holder for this preprint (which was not certified by peer review) is the author/funder, who has granted bioRxiv a license to display the preprint in perpetuity. It is made available under aCC-BY 4.0 International license.

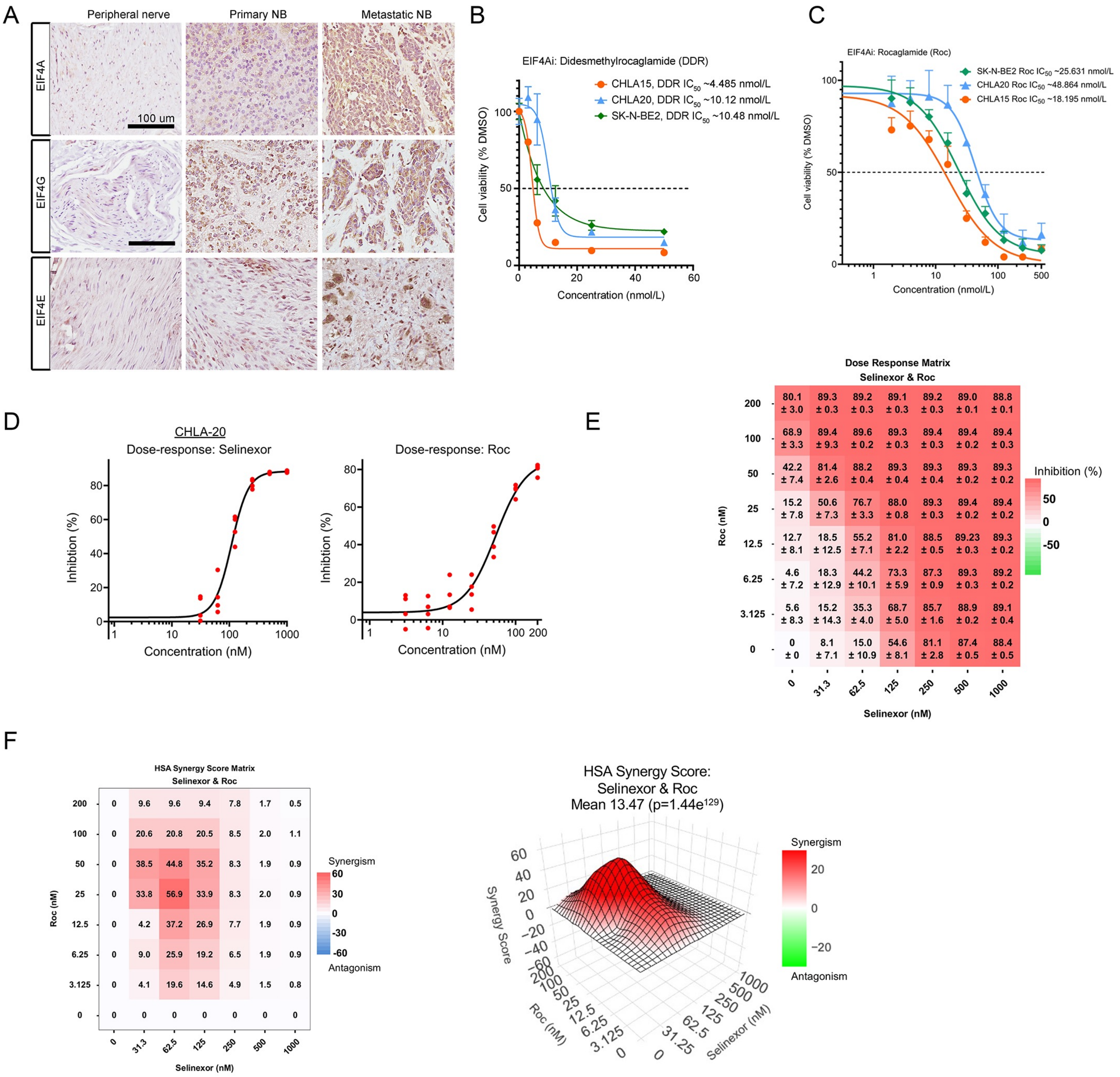

Figure S9
